# Management Strategies for Brain Tumors Diagnosed during Pregnancy: A Case Report and Literature Review

**DOI:** 10.3390/medicina57060613

**Published:** 2021-06-12

**Authors:** Reona Shiro, Kosuke Murakami, Masaharu Miyauchi, Yasuhiro Sanada, Noriomi Matsumura

**Affiliations:** 1 Department of Obstetrics and Gynecology, Kindai University Faculty of Medicine, Osakasayama 589-8511, Japan; reonashiro@med.kindai.ac.jp (R.S.); noriomi@med.kindai.ac.jp (N.M.); 2 Department of Neurosurgery, Kindai University Faculty of Medicine, Osakasayama 589-8511, Japan; msha3ru@med.kindai.ac.jp (M.M.); ysanada@med.kindai.ac.jp (Y.S.)

**Keywords:** brain tumor, choroid plexus papilloma, pregnancy, termination

## Abstract

*Background and Objectives*: Maternal brain tumors diagnosed during pregnancy are very rare, and their clinical course remains incompletely understood. We recently experienced a case of a brain tumor diagnosed at 30 weeks of gestation, and the treatment was initiated after delivery at 32 weeks of gestation. In this study, we reviewed case reports of brain tumors diagnosed during pregnancy, focusing on whether the brain tumor was treated during pregnancy or after termination of pregnancy and on the timing of therapeutic intervention. *Materials and Methods*: We searched PubMed and Ichushi-Web for articles published after January 2000 that reported cases of maternal brain tumors diagnosed during pregnancy. The patients were divided into two groups according to whether the tumor was treated during pregnancy (Group A) or after termination of pregnancy (Group B). *Results*: In total, 42 patients were included in the study (13 (31%) in Group A and 29 (69%) in Group B). The most common symptoms before diagnosis were those caused by increased intracranial pressure (57.1%). The diagnosis was made at 18 ± 6 weeks of gestation in Group A and 26 ± 9 weeks of gestation in Group B (*p* = 0.007). In all cases diagnosed after 34 weeks of gestation, termination of pregnancy was followed by treatment. Treatment was initiated within two weeks of diagnosis in 50% of patients in Group A and 30% in Group B. *Conclusions*: When severe symptoms caused by increased intracranial pressure last for several weeks, imaging tests should be considered. Termination of pregnancy is a good option for a brain tumor diagnosed after 34 weeks of gestation, while comprehensive treatment decisions should be made based on the severity of symptoms and the course of pregnancy in other cases.

## 1. Introduction

Maternal brain tumors diagnosed during pregnancy are very rare. A brain tumor itself can be life-threatening [1], and the condition of both the mother and fetus must be considered during pregnancy. Because of the low prevalence of such tumors, details regarding the diagnosis and treatment of brain tumors diagnosed during pregnancy remain incompletely understood. In particular, there is a lack of evidence on whether brain tumors should be treated during pregnancy or after termination of pregnancy, as well as on the timing and mode of delivery.

We recently experienced a case in which a maternal brain tumor was diagnosed at 30 weeks of gestation, and the tumor was treated after delivery at 32 weeks of gestation. In addition to reporting the course of this case, we conducted a literature review of similar cases of maternal brain tumors diagnosed during pregnancy and herein discuss the details of the clinical course.

## 2. Case Presentation

**Figure 1:**
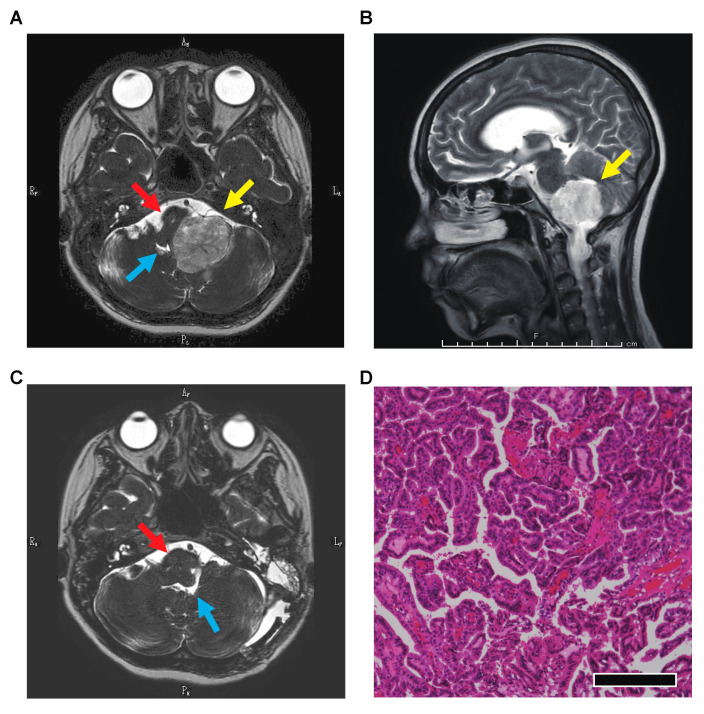
Magnetic resonance imaging of the brain and a specimen of the tumor. Imaging confirmed a mass (yellow arrow) on the left cerebellopontine angle compressing the brainstem (red arrow) and nearly obstructing the fourth ventricle (blue arrow). (**A**) T2-weighted image, horizontal section. (**B**) T2-weighted image, sagittal section. (**C**) T2-weighted image 14 days after the tumor resection, horizontal section. (**D**) A photomicrograph of a surgical specimen showing a papillary structure covered with a single layer of columnar epithelial cells along a narrow interstitium with abundant vessels. Hematoxylin and eosin stain, ×100, with a scale bar of 50 μm.

A 23-year-old woman (gravida 3 para 2) with no significant medical history or family history was referred to our hospital at 30 weeks of gestation because of nausea, vomiting and general fatigue. These symptoms had first appeared at 28 weeks of gestation, and gait imbalance and difficulty with speech also occurred a few days before referral. On examination, her Glasgow Coma Scale score was E3V5M6, and she exhibited a tremor in her left upper limb. She had experienced repeated nausea and vomiting, which had led to 3 kg of body weight loss within one month. Her vital signs were normal, and laboratory data showed no significant changes. Because the symptoms had lasted for two weeks and were accompanied by a change in her level of consciousness, a neurological disorder was suspected. A brain MRI was performed to search for an intracranial lesion, and a mass was found on the left cerebellopontine angle nearly obstructing the fourth ventricle (Figure 1A,B). Although the patient was prescribed steroids and osmotic diuretics, her symptoms caused by increased intracranial pressure remained unchanged. Obstetricians and neurosurgeons discussed her condition, which was considered to have a potential risk of obstructive hydrocephalus, and decided that the patient’s pregnancy should be terminated to proceed with treatment of the brain tumor. Cesarean section under general anesthesia was performed at 32 weeks of gestation, and the patient delivered a 1532-g female infant with an Apgar score of 4/7. One week after delivery, total removal of the tumor was conducted. A lateral suboccipital craniotomy was performed, with the dura incised along the sigmoid sinus. The tumor was detected after opening the lateral cerebellomedullary cistern. The floccular part of the fourth ventricle was assumed to be the tumor’s origin as the tumor adhered to that part. Total resection of the tumor was accomplished, which was confirmed by the postoperative brain MRI (Figure 1C). She was discharged from the hospital 17 days after the operation with no complications. Pathologic examination revealed a papillary structure covered with a single layer of columnar epithelial cells along a narrow interstitium with abundant vessels, which concluded the pathological diagnosis was choroid plexus papilloma (Figure 1D). No recurrence of the tumor had been seen for 18 months after the operation.

## 3. Materials and Methods

We conducted a literature search of case reports or case series on maternal brain tumors diagnosed during pregnancy using PubMed and Ichushi-Web (Japan Medical Abstracts Society), an article search engine mainly in Japanese. The query terms in PubMed were “pregnancy” AND “brain tumor” NOT review NOT meta-analysis, and the query terms in Ichushi-Web were the same words in Japanese. Considering the advancements that have been made in head computed tomography (CT) and magnetic resonance imaging (MRI) as diagnostic methods, cases were limited to those reported after 2000. Based on the title and abstract, cases of maternal brain tumors diagnosed during pregnancy were selected for our case review. Since we had confronted the challenging question in our case as to when treatment of a maternal brain tumor should be prioritized over fetal growth, the patients were divided into two groups according to the timing of treatment: those treated for brain tumors during pregnancy and those treated for brain tumors after termination of pregnancy. (In this review, “termination of pregnancy” refers to artificial or spontaneous termination of pregnancy regardless of gestational weeks.) The following information was extracted: age, past parity, number of gestational weeks at diagnosis and at termination of pregnancy, tumor size at diagnosis, first symptoms before diagnosis, histological type of a tumor, World Health Organization grade of tumor, site of a tumor, treatment of a tumor, mode of delivery and anesthetic technique during cesarean section.

The statistical analysis was carried out using GraphPad Prism 9.0.0 (GraphPad Software, San Diego, CA, USA).

## 4. Results

**Figure 2:**
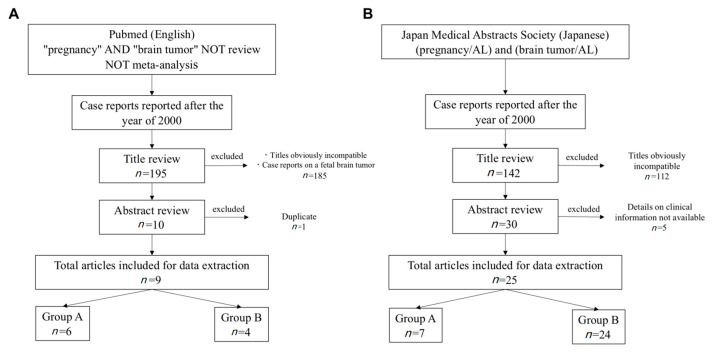
Literature search of PubMed and Ichushi-Web (Japan Medical Abstracts Society). (**A**) Literature search by PubMed. (**B**) Literature search by Ichushi-Web. The date of the last search was 30 July 2020. The search in Japanese was conducted by substituting English terms with Japanese synonyms.

Eight articles (11 cases) [2,3,4,5,6,7,8,9] were extracted from PubMed (Figure 2A), and 25 articles (30 cases) [10,11,12,13,14,15,16,17,18,19,20,21,22,23,24,25,26,27,28,29,30,31,32,33,34] were extracted from Ichushi-Web (Figure 2B). Thus, 42 cases including our case were reviewed. With respect to the treatment strategy, 13 (31%) patients were treated for brain tumors during pregnancy (Group A) and 29 (69%) patients were treated following termination of pregnancy (Group B). The mean age of the patients was 32 ± 4.7 years in Group A and 30 ± 5.4 years in Group B, with no significant difference between the two groups (Table 1). No significant difference in past parity or tumor size at diagnosis was present between the two groups (Table 1). The most common initial symptoms before diagnosis were those caused by increased intracranial pressure, such as headache, nausea and vomiting, accounting for 57.1% of symptoms in all patients; these were followed by seizure in 19.0% of patients and diplopia in 9.5% (Table 1).

**Figure 3:**
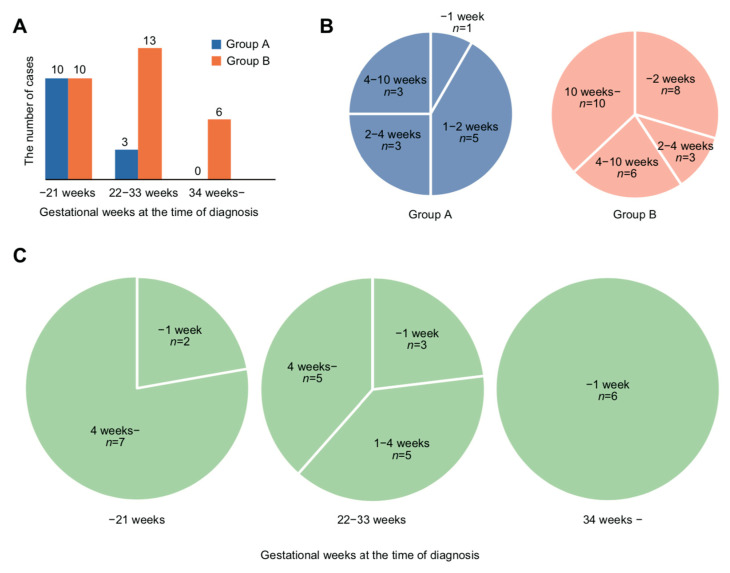
Gestational weeks at diagnosis of brain tumors and the time between diagnosis and initiation of treatment. (**A**) Number of cases in which treatment of the tumor was conducted during pregnancy (Group A) or after termination of pregnancy (Group B). Cases were divided into three groups according to the timing of diagnosis: before 21 weeks, 22 to 33 weeks and after 34 weeks of gestation. The numbers of cases in which patients were treated during pregnancy (Group A) and treated postpartum (Group B) are indicated by blue and orange, respectively. (**B**) Time between diagnosis and initiation of treatment of the tumor in Group A and Group B. The time between diagnosis and treatment in each group is illustrated in a pie graph. Three cases were excluded because data were unavailable. (**C**) Time between diagnosis and termination of pregnancy according to the timing of diagnosis in Group B. The time between diagnosis and termination of pregnancy was divided into three groups according to the timing of diagnosis: before 21 weeks, 22 to 33 weeks and after 34 weeks of gestation. The number of cases is shown in a pie graph. One case was excluded because data were unavailable.

Mesenchymal non-meningothelial tumors (23.8%), diffuse astrocytic and oligodendroglial tumors (21.4%), and meningiomas (19.0%) were the most common histological types of brain tumors (Table 1). The histopathological subdivision showed a high frequency of meningiomas overall (Group A, 15.4%; Group B, 20.7%) and a high frequency of hemangioblastomas in Group B (6 cases, 20.7%) (Table 1). Grade I tumors according to the 2016 World Health Organization classification of tumors of the central nervous system [35] accounted for the majority of tumors (about 70%), and grade II–IV tumors were also found in about 10% of patients in each group (Table 1). The mean number of gestational weeks at diagnosis of the brain tumor was 18 ± 6 in Group A and 26 ± 9 in Group B, with a significant difference between the two groups (*p* = 0.007) (Table 1). There was no difference in the number of cases diagnosed before 22 weeks of gestation (10 cases in both Group A and Group B). However, the number of cases in Group B increased as pregnancy progressed. In fact, among all cases diagnosed after 34 weeks of gestation, termination of pregnancy was followed by treatment (Figure 3A). In Group A, the shortest time from diagnosis to treatment of the tumor was five days, and the longest was nine weeks two days. In six (50%) of the 12 cases in which information on the timing of treatment was available, the patients received treatment within two weeks of diagnosis (Figure 3B). In Group B, treatment was started within two weeks of diagnosis in eight (30%) patients (Figure 3B). There was no significant difference in the timing of termination of pregnancy between the two groups (Table 1). With respect to the mode of delivery, cesarean section was selected in 79.5% of the patients. Of the 19 cases in which information on anesthesia during cesarean section was available, 16 (84.2%) patients underwent general anesthesia (Table 1). Three cases of artificial abortion occurred because of the priority of treatment (Table 1).

## 5. Discussion

Brain tumors can be found in young women, but the incidence of brain tumors in women of childbearing age is reportedly only 2.0–3.2 per 100,000 women [36]. Because of the low frequency of the disease, there is no confirmed evidence on the diagnosis and treatment of such tumors. Although a systematic review of case series on brain tumors in pregnancy was recently reported, the review included cases reported more than 20 years ago, thus likely yielding differences in the management of pregnancy as well as the diagnosis and treatment of the brain tumors [37]. In this study, we focused on cases reported mainly in Japan after the year 2000 in an attempt to analyze patients with similar medical backgrounds. Moreover, this is the first report to evaluate the treatment of a tumor in terms of the timing of pregnancy, with particular attention to whether the brain tumors were treated during pregnancy or after termination.

It is quite difficult to diagnose a brain tumor based on the initial symptoms [38]. Generally, the symptoms of brain tumors are often associated with increased intracranial pressure [38]. In this study, including the case we experienced, more than half of the initial symptoms that triggered the diagnosis were those caused by increased intracranial pressure such as headache, nausea and vomiting. Among these symptoms, headache is a common symptom in pregnancy, and nausea and vomiting also occur with hyperemesis gravidarum and uterine enlargement [36,39]. Therefore, when a pregnant woman complains of these symptoms, it is necessary to consider their duration, severity and associated symptoms to avoid overlooking the possibility of a brain tumor. Another factor contributing to this challenging diagnosis is that doctors tend to avoid imaging tests during pregnancy because of the risk of radiation exposure. As long as contrast media are not used, the risk to the fetus during head CTs and MRIs is negligibly low [40]. Therefore, clinicians should not hesitate to perform these imaging tests when necessary.

In this study, the most common histological type of tumor was meningioma (19.5%, eight cases). This may be related to the fact that meningiomas are known to express progesterone receptors, which lead to increased symptom severity during pregnancy [41]. The histological types of brain tumors are diverse [38], and in this study, we found a great variety of histological types not necessarily specific to pregnancy, which is in accordance with previous reports [37,42].

The most challenging question regarding the treatment of brain tumors diagnosed during pregnancy is whether the brain tumor should be treated during pregnancy or after termination of pregnancy. If the brain tumor is treated during pregnancy, continuation of pregnancy will allow the fetus to mature; however, the treatment might adversely impact the pregnancy [36]. In particular, it is difficult to treat brain tumors during pregnancy if treatment requires surgical positions that are difficult to obtain during pregnancy [43], or chemotherapy or radiation therapy [36]. Conversely, if the treatment is performed after termination of pregnancy, consideration of its effect on the pregnancy can be ignored; however, the impact of preterm birth on the infant must be considered [44]. In this study, we found that when the brain tumor was diagnosed later in pregnancy, the patient was more likely to receive treatment for the brain tumor after termination of pregnancy (Figure 3A). In fact, when the diagnosis was made at 34 weeks or later, all patients underwent termination of pregnancy first followed by treatment of the brain tumor after delivery (Figure 3A). Moreover, in all patients diagnosed with brain tumors at 34 weeks or later, termination of pregnancy was conducted within ≤1 week (Figure 3C). Currently, in many countries, administration of steroids to the mother is recommended to promote pulmonary maturation and prevent intracranial hemorrhage in the infant when preterm birth is expected within one week after 24 weeks and before 34 weeks of gestation. This recommendation leads to the idea that 34 weeks of gestation is one of the criteria for ensuring the postnatal safety of the infant [45,46]. In addition, the incidence of neonatal complications in preterm infants decreases after 32 weeks of gestation [47], which is a reasonable indicator of when to consider termination of pregnancy in favor of the maternal condition.

In half of the patients in this study, however, treatment was started within two weeks of diagnosis. The reason for the rapid initiation of treatment despite the prematurity of the fetus was that the maternal condition deteriorated because of the worsening intracranial pressure or emerging symptoms caused by compression of the brain, and the benefits to the fetus by continuing the pregnancy took precedence over the disadvantages to the fetus caused by therapeutic intervention.

The grading of the tumors in this study was similar to that of previous reports of non-pregnant cases [48]. Notably, intensive treatment may be urgently needed depending on the tumor grade [49]. In this study, there were two patients with grade IV tumors in both Group A and Group B (Table 1). In Group A, the pregnancies were terminated at 29 and 32 weeks of gestation, respectively, to allow for postoperative adjuvant chemotherapy. Of the two patients in Group B, one underwent an artificial abortion before 22 weeks, and the other was diagnosed with a brain tumor at 20 weeks and eventually underwent termination of pregnancy at 29 weeks of gestation to proceed with treatment of the tumor. Thus, it is necessary to explain to the patient that early termination may be necessary if a malignant tumor is suspected.

With respect to the mode of delivery, cesarean section was selected in most patients, which is consistent with previous reports [37]. Cesarean section is an option to avoid increased intracranial pressure due to vaginal delivery [39]. Cesarean section may also be chosen because of the difficulty of vaginal delivery due to immaturity of the cervix in preterm labor. Although there is no confirmed evidence on the safety of anesthesia according to technique (general or regional) in patients with brain tumors undergoing cesarean section, general anesthesia is recommended for patients with signs of increased intracranial pressure to avoid regional anesthesia because of the risk of cerebral herniation [39,50].

There was no significant difference in the timing of termination of pregnancy between the two groups (Table 1). Four of eight cases of cesarean sections and one of two cases of vaginal deliveries in group A were preterm due to the urgent need of termination of pregnancy for the further treatment of the mother. Even though interventions for the tumor were conducted during pregnancy for growth and maturation of the fetus, pregnancy in half of those cases eventually had to be terminated because of deterioration of maternal conditions or postoperative adjuvant chemotherapy. We believe the number of cases ending up in preterm deliveries in group A contributed to the lack of a significant difference between the two groups.

This study was a literature review focusing on case reports of a rare condition with a small number of total cases, which potentially yields a risk of publication bias. On the other hand, about 75% of the cases were diagnosed and treated in Japan, providing the advantage of a relatively universal clinical background. We believe that it is extremely important to achieve an overview of maternal brain tumors during pregnancy by further accumulation of cases because the establishment of management strategies for both the mother and fetus is very challenging.

## 6. Conclusions

When obstetricians encounter pregnant patients complaining of symptoms caused by increased intracranial pressure such as headaches, nausea and vomiting, especially when the symptoms are severe and last for several weeks, a brain CT or MRI should be considered to search for intracranial lesions. When a brain tumor is diagnosed after 34 weeks of gestation, termination of the pregnancy is a good option to allow for treatment of the mother. In other cases, decision-making depends largely on the severity of symptoms caused by the tumor and the time course of the pregnancy.

## Figures and Tables

**Table 1:** The backgrounds and clinical findings of patients in the reviewed cases.

		Group A	Group B	%	*p*-Value
		*n* = 13	*n* = 29		
Age		32 ± 4.7	30 ± 5.4		0.18
Parity					0.46
	primipara	7	13		
	multipara	3	12		
Timing of diagnosis (gestational weeks)		18 ± 6	26 ± 9		0.007
Timing of termination of pregnancy (gestational weeks)		35 ± 4	33 ± 7		0.28
Size of a tumor on diagnosis (cm)		4.3 ± 2.0	4.1 ± 1.4		0.67
Initial symptoms before diagnosis					
	headache, nausea and vomiting	7	17	57.1	
	seizures	3	5	19.0	
	impaired vision	1	3	9.5	
	others	2	4	14.3	
Histological type of tumor					
mesenchymal, non-meningothelial tumors		1	9	23.8	
	hemangioblastoma	1	6		
	others	−	3		
Diffuse astrocytic and oligodendroglial tumors		3	6	21.4	
	diffuse astrocytoma	−	2		
	glioblastoma	2	2		
	others	1	1		
	N/A	−	1		
Meningiomas (Meningioma)		2	6	19.0	
Neuronal and mixed neuronam-glial tumors		1	2	7.1	
Tumors of the sellar region		1	2	7.1	
Other astrocytic tumors (pilocytic astrocytoma)		−	1	2.4	
Ependymal tumors (anaplastic ependyoma)		1	−	2.4	
Schwannoma		−	1	2.4	
Choroid plexus tumors (choroid plexus papilloma)		−	1	2.4	
N/A		4	1	11.9	
Grading of tumors according to the 2016 CNS WHO					
Ⅰ		5	19	57.1	
Ⅱ		−	3	7.1	
Ⅲ		2	2	9.5	
Ⅳ		2	2	9.5	
The site of a tumor at diagnosis					
Frontal lobe		5	7	28.6	
Parietal lobe		1	4	11.9	
Cerebellum		1	6	16.7	
Sellar region		1	2	7.1	
Cerebral ventricle		2	−	4.7	
Others		3	7	23.8	
N/A		−	3	7.1	
Treatment					
Craniotomy		8	15	56.1	
Embolization → craniotomy		−	5	12.2	
Craniotomy → radiation		2	3	12.2	
Craniotomy → chemoradiation		−	3	7.3	
Resection of a tumor via nasal approach		1	1	4.9	
Radiation		1	−	2.4	
Radiosurgery		1	−	2.4	
Chemoradiation		−	1	2.4	
Cesarean section or vaginal delivery (after 22 weeks of gestation)					
Cesarean section		8	23	79.5	
	under general anesthesia	2	14		
	under spinal anesthesia	1	2		
	N/A	5	7		
Vaginal delivery		2	3	12.8	
Artificial abortion		−	3	7.7	

N/A: not available; CNS: central nervous system; WHO: World Health Organization.

## Data Availability

The datasets used and/or analyzed during the current study are available from the corresponding author on reasonable request.
